# Postural fall in systolic blood pressure is a useful warning sign in dengue fever

**DOI:** 10.12688/f1000research.132714.2

**Published:** 2023-12-18

**Authors:** Chakrapani Mahabala, Archith Boloor, Sushmita Upadhya, Satya Sudish Nimmagadda, Tejaswini Lakshmikeshava, Raghav Anand

**Affiliations:** 1 Medicine, Kasturba Medical College , Mangalore , Manipal Academy Of Higher Education, Manipal, Karnataka, 575001, India; 2 Cardiology, Andhra Medical College, Vishakapatanam, Andhra Pradesh, 530002, India

**Keywords:** Severe Dengue, hematocrit, Warning signs, Postural Hypotension, Hemoconcentration, Thrombocytopenia

## Abstract

**Background:**

Capillary leak is the hallmark of development of severe dengue. A rise in haematocrit has been a major warning sign in WHO guidelines. Postural hypotension, which could reflect the intravascular volume reduction in capillary leak has been noted as warning sign in CDC and Pan American Health Organisation guidelines. We evaluated the diagnostic accuracy of postural hypotension as a marker of development of severe dengue.

**Methods:**

150 patients admitted with dengue fever were recruited in this prospective observational study. Diagnostic accuracy of conventional warning signs (abdominal pain, persistent vomiting, fluid accumulation, mucosal bleeding, lethargy, liver enlargement, increasing hematocrit with decreasing platelets) and postural hypotension was evaluated.

**Results:**

23 (15.3%) subjects developed severe dengue. Multiple logistic regression analysis showed that ascites/pleural effusion and postural fall in systolic blood pressure of >10.33% had odds ratio of 5.024(95%CI:1.11 – 22.75) and 11.369 (95% CI:2.27 – 56.87), respectively. Other parameters did not reach statistical significance. Sensitivity and specificity of ascites/pleural effusion were 82.6% and 88.2% for development of severe dengue whereas postural fall in systolic blood pressure had sensitivity and specificity of 87% and 82.7%.

**Conclusions:**

These findings present a strong case for including postural hypotension as a warning sign in patients with dengue fever, especially in resource limited settings.

## Introduction

Mosquito-borne dengue viral fever is endemic to India. Every year thousands of cases are detected throughout India despite mosquito control measures. ^1^ In 2020, India recorded 44585 cases of dengue and in 2021, it increased to 123106 cases. ^1^ Dengue progresses from a stage of fever without warning signs to dengue with warning signs and then to severe dengue. About 5-15% of patients with dengue fever progress to severe dengue. ^2^ This febrile illness has the potential to transform into severe dengue and result in mortality, even in young healthy population. ^3^

It’s clinically difficult to identify those patients with dengue fever who later on progress to severe dengue. ^3^^,^^4^ World Health Organisation has identified a set of seven clinical and laboratory parameters as warning signs (abdominal pain, persistent vomiting, fluid accumulation, mucosal bleeding, lethargy, liver enlargement, increasing hematocrit with decreasing platelets) which are extensively used in clinical practice for early identification of progression to severe dengue. ^2^ There have been many studies evaluating the diagnostic accuracy of these parameters in dengue fever with mixed results. ^2^^,^^5^^–^^7^ In general these parameters have high specificity and high negative predictive value but a lower sensitivity and lower positive predictive value. ^2^^,^^7^

Capillary leak is the hallmark of onset of severe dengue. In clinical practice, capillary leak is identified by hemoconcentration, a significant rise in hematocrit, and evidence of fluid collection in cavities. Rise in hematocrit and concurrent significant drop in platelet count is an important warning sign. To identify this parameter, patients are subjected to repeated blood investigations. Availability of good laboratories, manpower, and money for repeating these tests frequently would be a challenge in rural areas. Moreover, interpretation of rising hematocrit requires a baseline hematocrit value which is unavailable in most of the patients when they get admitted in resource-limited settings. ^8^^,^^9^ Assuming baseline hematocrit with population data is advised when baseline values are not available. ^4^ This is not reliable, because of the wide variability of hemoglobin and hematocrit in third-world countries. It also was noted earlier that many patients did not have a significant rise in hematocrit even when they develop shock in dengue fever. ^10^

Capillary leak leads to hypovolemia and varying degree of hypotension. ^11^ Most of the clinical manifestations of dengue complications can be attributed to capillary leak syndrome. ^11^ Orthostatic blood pressure changes are commonly used to assess intravascular volume. ^12^ Hence, postural fall in blood pressure could be an indicator of significant capillary leak in early part of dengue fever. CDC guidelines and Pan American Health Organisation (PAHO) guidelines include Postural hypotension as one of the warning signs. ^13^^,^^14^ Clinical criteria used in dengue management at the Queen Sirikit National Institute of Child Health, Bangkok also include postural hypotension as one of the parameters for admission to the hospital. ^15^ Postural hypotension is not included as warning sign in WHO guidelines. Conventional warning signs (defined by WHO) have been studied extensively as predictors of severe dengue. However, data regarding postural hypotension in this condition is very limited. Hence this study was planned to evaluate the accuracy of postural hypotension in identifying patients with severe dengue and develop a simple model for identifying the subsequent development of severe dengue.

## Methods

### Study design and sample

Patients admitted to hospital with dengue fever as per WHO criteria between 2011-2015 were included in this prospective observational study. Patients of 18 years and above with NS1 antigen positive results or positive dengue IgM ELISA report were included. Patients with severe dengue on admission were excluded. In our study, to avoid bias, inclusions, measurements and outcomes were all objective. Descriptive clinical data regarding atypical manifestations of dengue among these patients was published earlier. ^16^ Analytical data regarding diagnostic performance of warning clinical signs and postural hypotension are presented in this paper. Patients with pre-existing postural hypotension were excluded.

### Sample size

We calculated the sample size by assuming the expected prevalence to be 15.3%, based on a previous study published by Alok kumar *et al.*, ^3^ with a sensitivity of 70% and specificity of 85%, precision of 20% and 95% confidence interval and a drop out percentage of 5%. The sample size was estimated as 143.

### Variables and procedure

Complete blood count (by pulse detection/fluorescence flow cytometry using Sysmex XN 9000), erythrocyte sedimentation rate (by automated using Sysmex XN9000), liver function test (by 3,5-dichlorophenyldiazonium tetrafluoroborate (DPD) for bilirubin, biuret test for proteins/UV Kinetic colorimetric method for Alanine transaminase and Aspartate transaminase using Cobas Pro.), creatinine (by Jaffe colorimetric using Cobas Pro; the Cobas Pro analyzer series is automated system using a combination of photometric and ion-selective electrode (ISE) determinations, and electrochemiluminescence (ECL) signal in the immunoassay analysis module (e601 module), chest X ray, abdominal ultrasound were done on admission. Hemoglobin, hematocrit and platelet count was done daily for the next 48 hrs.

The sample was drawn by the nurse in the respective wards (2cc of EDTA and 2 cc of serum sample). The samples were analysed in the central laboratory by automated machines as described. The results were ratified by the laboratory incharge faculty.

Rise in hematocrit was calculated by comparing the hematocrit after admission with population mean values. ^4^ Hematocrit was measured by pulse detection method using the Sysmex XN analyser 9000. Mean baseline hematocrit for South Indian males was 44.3% and for females 36.4%. ^17^ Rise of 20% compared to the baseline hematocrit was considered significant (53.2% for males and 43.7% for females).

Patients were treated as per the WHO 2009 Protocol for the management of Dengue. Patients were followed up until they recovered or succumbed to the illness. Patients who developed severe dengue were identified as per the WHO criteria. ^18^

### Ethical considerations

Ethical committee clearance was obtained from the institutional ethics committee at Kasturba Medical College, Mangalore, India (approval number – IEC KMC MLR 03/2022/82). Written informed consent was obtained from the participants for collection of data, analysis of data and publication of findings.

### Data analysis

Data was analysed using the software SPSS version 25 (RRID:SCR_002865). Sensitivity, specificity, negative predictive value, and positive predictive value were calculated for all 7 warning signs and postural fall in systolic blood pressure (SBP). Multiple logistic regression test was performed to find out the adjusted odds ratio of parameters between non-severe and severe dengue patients. Receiver operator characteristic curve analysis was done to find the optimal cut-off value of the continuous variables which would yield the best specificity and sensitivity for severe dengue fever. Based on the logistic regression analysis, parameters that had significant odds ratio were identified and decision tree analysis was performed using those parameters as independent variables and severe dengue as the dependent variable.

CHAID method of model development was used to develop the decision tree model with 70% of the sample for development of the model (training sample) and 30% of the data (test sample) for split sample validation of the model. Accuracy of the model for training and test sample was expressed as accuracy with a 95% confidence interval. Specificity, sensitivity, negative likelihood ratio, positive likelihood ratio, and diagnostic odds ratio were calculated for the entire dataset using the model developed by the decision tree.

## Results

Clinical and laboratory data of 150 dengue patients admitted to the hospital were analysed. ^22^ All 150 subjects who were included were available for evaluation till the end of study except for one patient who succumbed to the illness. 74% were men and 26% were women. The mean age of the subjects was 37.9±15.3 years. 23 patients with dengue developed severe dengue as per the World Health Organisation classification. The median duration of fever before admission was four days (IQ range 3–6). There was a statistically significant difference in hemoglobin, haematocrit, and postural fall in the systolic blood pressure (SBP) on admission between the groups which developed severe dengue subsequently and the group which remained in the non-severe category ( Table 1).

**Table 1.** Difference in parameters between the groups with severe dengue and non-severe dengue.

	Severe (Mean values with standard deviation)	Non-Severe (Mean values with standard deviation)	p value
Age (years)	31.22 (10.89)	39.14 (15.69)	0.022
Duration of fever before admission (days)	4.43 (1.61)	4.61 (1.9)	0.671
Hemoglobin on admission (g/dL)	15.88 (2.52)	14.42 (1.78)	0.001
Haematocrit on admission (%)	45.67 (6.60)	41.97 (4.28)	0.001
Platelet count on admission (cells/μL)	46087 (53715)	74724.40 (57778)	0.029
Total leukocyte count (cells/μL)	6433.48 (2762.11)	4902.28 (2697.52)	0.014
Aspartate transaminase (IU/mL)	802.04 (1587.50)	200.13 (219.07)	<0.001
Lowest platelet (cells/μL)	23304 (10881)	53417 (40419)	0.001
Percentage fall in SBP (%)	14.83 (4.39)	8.36 (4.5)	<0.001
Albumin	3.54 (0.33)	3.74 (0.43)	0.042

Warning signs showed good specificity and positive predictive value for severe dengue, but in general, sensitivity and negative predictive values were low, except for pleural effusion/ascites which had good sensitivity and specificity. Postural fall in SBP also showed higher sensitivity and specificity ( Table 2). Patients developing postural hypotension during the stay in the hospital were also included in the group with postural hypotension if they did not have clinical and laboratory features of severe dengue.

**Table 2.** Sensitivity, specificity, negative predictive value and positive predictive value of warning signs and postural fall in systolic blood pressure.

Parameter	Sensitivity (%)	Specificity (%)	Negative predictive value (%)	Positive predictive value (%)
Rise in Hematocrit	13	96.9	86.0	42.8
Ascites/Pleural Effusion	82.6	88.2	96.6	55.9
Postural Fall in Systolic Blood Pressure	87	82.7	97.2	47.6
Hepatomegaly	30.4	91.3	87.9	38.9
Lethargy	4.3	99.2	85.1	15.3
Persistent Vomiting	65.2	64.6	91.1	25.0
Severe abdominal Pain	82.6	66.1	95.5	30.6
Mucosal bleeding	60.9	89.0	92.6	50.0

Receiver operator curve (ROC) analysis showed an area under the curve of ROC 0.86; p<0.001 (95% CI: 0.77-0.95) and analysis of the coordinates showed that postural fall of more than 10.33% in systolic BP was the ideal cut-off. Multiple logistic regression analysis revealed that except for postural fall in the SBP on admission and the presence of pleural effusion/ascites, none of the other parameters had a statistically significant adjusted odds ratio ( Table 3).

**Table 3.** Multiple logistic regression analysis to identify parameters with a statistically significant adjusted odds ratio.

Parameter	Wald	Adjusted odds ratio	95% CI for odds ratio	Significance (p)
Rise in Hematocrit	.484	2.151	0.25 – 18.63	.487
Ascites/pleural Effusion	4.400	5.024	1.11 – 22.75	.036
Postural Fall in Systolic BP	8.759	11.369	2.27 – 56.87	.003
Hepatomegaly	.436	1.753	0.33 – 9.27	.509
Lethargy	.139	5.325	0.001-35073	.709
Persistent Vomiting	.925	2.034	0.479 – 8.64	.336
Severe Abdominal Pain	2.396	3.182	0.74 – 13.78	.122
Mucosal Bleeding	3.682	3.865	0.97 – 15.38	.055

Hence pleural effusion/ascites and postural fall in the systolic BP were taken as independent parameters for the decision tree analysis. Results of decision tree analysis showed that the model had a very high accuracy of 93.7% (95% CI: 88.9-98.5) for the training data set and 86.7% (95% CI: 76.5-96.9) for the test data set (validation data set). The cross-tabulation of observed and predicted values is mentioned in Table 4.

**Table 4.** Comparison of model predication and actual status.

		Severe Dengue	Non-Severe Dengue
Prediction by Model	Severe Dengue	18	8
	Non-Severe Dengue	5	119

**Figure 1.**
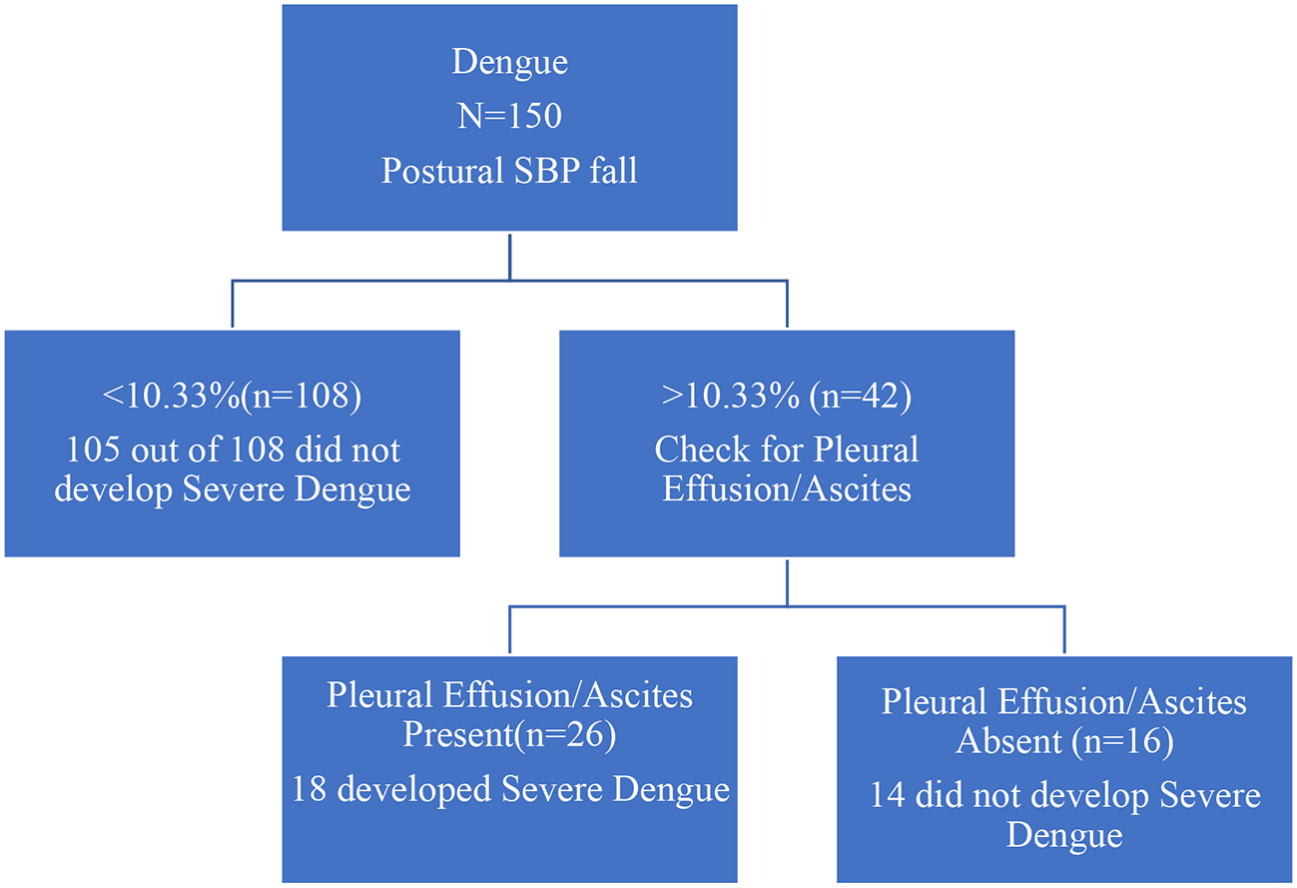
Decision tree for detecting cases at risk of progression to severe dengue.

Patients with less than 10.33% fall on standing SBP were very unlikely to develop severe dengue than those with more than 10.33% fall in the systolic BP. Patients who did not have pleural effusion/ascites were also unlikely to develop severe dengue ( Figure 1).

Specificity, sensitivity, negative likelihood ratio, positive likelihood ratio, negative predictive value, positive predictive value, and diagnostic odds ratio of the model for the entire data set is shown in Table 5.

**Table 5.** Diagnostic accuracy of the decision tree.

Parameter	Value
Sensitivity (%)	78.2
Specificity (%)	93.7
Positive Predictive Value (%)	69.2
Negative Predictive Value (%)	96.0
Positive Likelihood ratio:	12.4
Negative Likelihood ratio:	0.23
Diagnostic Likelihood ratio:	53.9

Three subjects succumbed to the illness and all of them had postural SBP fall of more than 10.33% and had pleural effusion/ascites in the initial screening and were found to have postural SBP fall of more than 18%.

## Discussion

We have studied 150 inpatients with dengue and evaluated the diagnostic performance of warning signs and postural hypotension. Fluid accumulation in cavities and postural fall in SBP of >10.33% were found to have good sensitivity and specificity. Multiple logistic regression showed that other warning signs did not have significant adjusted odds ratio for the development of severe dengue compared to ascites/pleural effusion and postural fall in SBP. Based on decision tree analysis, we developed a model to identify patients with high risk of progression to severe dengue utilizing simple clinical tools (postural hypotension and pleural effusion/ascites). Diagnostic odds ratio of the model is 53.9, making the decision tree a very good diagnostic tool for ruling out the risk of progression to severe dengue. This model performed well both in the development and validation cohort confirming good internal validity.

Severe dengue develops in about 5-15% of patients with dengue fever. ^2^ Capillary leak is a unique and major pathogenetic mechanism in the development of severe dengue. Hence, markers of capillary leak like rising hematocrit and fluid accumulation in cavities have been part of dengue management protocols. WHO has identified seven warning signs for severe dengue: abdominal pain, persistent vomiting, fluid accumulation, mucosal bleeding, lethargy, liver enlargement, increasing hematocrit with decreasing platelets. These markers have been used extensively to triage patients with dengue fever to identify those at higher risk of developing severe dengue. Severe dengue has high mortality of nearly 20% which can be brought down to nearly 1% of patients if identified early and managed appropriately. ^6^ Recently, many studies have been published evaluating the performance of these parameters in predicting severe dengue. In a study published from Malaysia, 700 patients were studied to evaluate the diagnostic performance of warning signs for association with severe dengue. Though specificity was good, sensitivity was suboptimal. Sensitivity of rise in hematocrit was 0.29 which was similar to our study (0.13) and specificity was above 90%. ^2^

Capillary leak occurs secondary to damage of endothelial cell-to-cell junction. It can occur as a primary event (Clarkson’s disease) or secondary to a variety of infections like dengue or other inflammatory conditions. ^11^ Capillary leak can lead to intravascular changes (hemoconcentration, hypovolemia- postural hypotension), extravascular changes (fluid accumulation in cavities) and organ involvement (abdominal pain, vomiting, lethargy, liver involvement). Intravascular changes have been typically assessed with hemoconcentration-rise in hematocrit. Importance of this phenomenon was identified around 1970 in Thailand and management protocols were developed by WHO subsequently based on this concept. ^15^ However, requirement of basal level to calculate rise in hematocrit is a major challenge in third world countries. Other parameters like postural hypotension would be an appropriate alternative to hematocrit in these situations since sensitivity and specificity for postural hypotension were very high for diagnosing severe dengue.

Evaluation of the rise in hematocrit as a predictor of severe dengue has yielded mixed results. In a meta-analysis of 87 studies consisting of 35,184 dengue fever and 8,173 severe dengue, 34 clinical/biochemical factors were associated with severe dengue out of which 9(including plasma leakage) were relevant within the 7 days window. ^6^ The odds ratio for pleural effusion for association with severe dengue was 15.83, p<001. Hematocrit was strongly associated with severe dengue (standardized mean difference=0.327, 95% CI: 0.019-0.546, p=0.003). ^6^ However, these observations were not confirmed in a meta-analysis involving a larger number of studies. ^5^ Sangkaew *et al.* performed a systematic review and meta-analysis of studies that focussed on predicting severe dengue using clinical and biochemical parameters during the febrile phase of the illness. ^5^ 150 research papers were included. Hemoconcentration was not found to be significantly different between the group which developed severe dengue compared to the group which remained non-severe. The standardised mean difference was 0.07 (95% CI: -0.11 to 0.26) which was statistically not significant (p= 0.59). ^5^ Moreover, 53.8% of patients with severe dengue did not have hemoconcentration during an epidemic of dengue in Brazil in 2008. ^7^ Difficulties in identifying plasma leakage by clinicians and the need for clinical parameters instead of laboratory parameters, especially in resource-limited settings, were also highlighted by Horstick *et al*. ^9^

There have also been a few studies in the past attempting to create a decision tree to help identify patients with a risk of progression to severe dengue, earlier on in the illness. ^19^^–^^21^ These studies either used biochemical parameters, had a lower diagnostic accuracy compared to the decision tree in the current study, or did not have an internal validation. A study by Tamibmaniam *et al*. tried to find the factors associated with severe dengue infection and to create a decision tree similar to our study. ^20^ Simple logistic regression analysis found many factors to have the ability to predict severity, but multiple logistic regression narrowed it down to pleural effusion, vomiting, and low systolic blood pressure. Using these variables, they created a decision tree with a sensitivity of 0.81, specificity of 0.54, PPV of 0.16, and NPV of 0.96. Our decision tree has a comparable sensitivity and NPV while having a much higher specificity and PPV. Also, the study by Tamibmaniam *et al*. did not include an internal validation of their decision tree, unlike in the current study where the internal validation provided by the training sample further validates the results of the test sample. WHO has developed a set of clinical warning Symptoms for triaging patients to decide inpatient management. These 7 parameters have been studied in various studies to identify the relative importance of each one of them in predicting severe dengue. Tamibmaniam *et al.* ^20^ have studied 657 patients with dengue of whom 59 had severe dengue. Even though many parameters were associated with severe dengue in simple logistic regression, adjusted odds ratios were significant for 3 parameters only after multiple logistic regression analysis (Fluid accumulation, vomiting and hypotension). Other parameters did not reach statistical significance in this multiple logistic regression. Our findings are also in line with these observations. It appears that some of the parameters seem to be having stronger independent association compared to other parameters. Our findings suggest that Postural fall in Systolic Blood Pressure and Pleural effusion/Ascites have much stronger independent associations with severe dengue, compared to other warning signs, resulting in relatively lower significance for other warning symptoms. Hence, these findings are unlikely to be due to bias.

Dengue patients can be easily and effectively triaged by checking for postural hypotension on admission and daily thereafter. If SBP fall is more than 10.33%, evaluation for pleural effusion/ascites can be done. Patients with pleural effusion/ascites and postural hypotension are the ones who need close monitoring and complete evaluation with a full battery of investigations as per WHO protocol to confirm the high risk of progression to severe dengue. Other patients may not require this aggressive approach since the model has a negative predictive value of about 94%. Postural hypotension represents intravascular changes and ascites/pleural effusion represents extravascular changes.

Identifying patients likely to develop severe dengue is a complex clinical decision-making process. Warning signs defined by WHO help the clinician in this process. No single parameter of these signs is definitive. Findings of this study confirm that postural hypotension is an important and useful warning sign in dengue fever. CDC and PAHO have included postural hypotension as one of the warning signs in their guidelines. ^13^^,^^14^ WHO guidelines do not include postural hypotension as warning sign. There is a strong case for inclusion of postural hypotension in other guidelines also. Our clinical decision tool could help clinicians in remote places make effective and appropriate clinical judgments when baseline hematocrit is unavailable.

### Limitations

The sample size is small. The results of our study need to be confirmed in bigger and multicentric settings to establish external validity.

## Data Availability

Dryad: Postural fall in Systolic Blood Pressure is an useful warning sign in Dengue Fever. https://doi.org/10.5061/dryad.jwstqjqfc. ^22^ Data are available under the terms of the Creative Commons Zero “No rights reserved” data waiver (CC0 1.0 Public domain dedication).

## References

[1] Directorate General of Health Services MoHaFW G of I 2022. Dengue cases and deaths in the country since 2015. [cited 2023 Feb 12]. Reference Source

[2] Ahmad MH Ibrahim MI Mohamed Z : The sensitivity, specificity and accuracy of warning signs in predicting severe dengue, the severe dengue prevalence and its associated factors. *Int. J. Environ. Res. Public Health.* 2018 Sep 15;15(9). 10.3390/ijerph15092018 30223572 PMC6163319

[3] Kumar A Mayers S Welch J : The spectrum of disease severity, the burden of hospitalizations and associated risk factors in confirmed dengue among persons of all ages: findings from a population based longitudinal study from Barbados. *Infect. Dis.* 2020;52(6):396–404. 10.1080/23744235.2020.1749723 32286109

[4] Wilder-Smith A Ooi EE Horstick O : Dengue. *Lancet.* 2019 Jan 26;393(10169):350–363. 10.1016/S0140-6736(18)32560-1 30696575

[5] Sangkaew S Ming D Boonyasiri A : Risk predictors of progression to severe disease during the febrile phase of dengue: a systematic review and meta-analysis. *Lancet Infect. Dis.* 2021 Jul 1;21(7):1014–1026. 10.1016/S1473-3099(20)30601-0 33640077 PMC8240557

[6] Yuan K Chen Y Zhong M : Risk and predictive factors for severe dengue infection: A systematic review and metaanalysis. *PLoS One.* 2022 Apr 1;17(4 April):e0267186. 10.1371/journal.pone.0267186 35427400 PMC9012395

[7] Cavalcanti LPG de Martins Mota LA Lustosa GP : Evaluation of the WHO classification of dengue disease severity during an epidemic in 2011 in the state of Ceará, Brazil. *Mem. Inst. Oswaldo Cruz.* 2014 Feb;109(1):93–98. 10.1590/0074-0276140384 24626308 PMC4005528

[8] Rodrigo C Sigera C Fernando D : Plasma leakage in dengue: a systematic review of prospective observational studies. *BMC Infect. Dis.* 2021 Dec 1;21(1):1082. 10.1186/s12879-021-06793-2 34670495 PMC8527656

[9] Horstick O Farrar J Lum L : Reviewing the development, evidence base, and application of the revised dengue case classification. *Pathog. Glob. Health.* 2012 May;106(2):94–101. 10.1179/2047773212Y.0000000017 22943544 PMC3408880

[10] Gupta P Khare V Tripathi S : Assessment of World Health Organization definition of dengue hemorrhagic fever in North India. 2010. 10.3855/jidc.708 20351455

[11] Siddall E Khatri M Radhakrishnan J : *Capillary leak syndrome: etiologies, pathophysiology, and management. Vol. 92, Kidney International.* Elsevier B.V.;2017; pp.37–46. 10.1016/j.kint.2016.11.029 28318633

[12] Kalantari K Chang JN Ronco C : *Assessment of intravascular volume status and volume responsiveness in critically ill patients. Vol. 83, Kidney International.* Nature Publishing Group;2013; pp.1017–1028. 10.1038/ki.2012.424 23302716

[13] Clinical Presentation|Dengue|CDC:[cited 2023 Feb 12]. Reference Source

[14] PAHO, WHO: Regional Arboviral Disease Program Algorithms for the Clinical Management of Dengue Patients. 2020.

[15] Kalayanarooj S Rothman AL Srikiatkhachorn A : Case management of dengue: Lessons learned. *J. Infect. Dis.* 2017;215:S79–S88. 10.1093/infdis/jiw609 28403440 PMC5853291

[16] Sudhish Nimmagadda S Mahabala C Boloor A : Atypical Manifestations of Dengue Fever (DF) - Where Do We Stand Today? *J. Clin. Diagn. Res.* 2014 Jan 12 [cited 2023 Feb 12];8(1):71–73. Reference Source 10.7860/JCDR/2014/6885.3960 PMC3939591 24596727

[17] Pasupula DK Reddy PS : When is a south Indian really anemic? *Indian J. Clin. Biochem.* 2014 Oct 1;29(4):479–484. 10.1007/s12291-013-0386-0 25298629 PMC4175707

[18] World Health Organization: *Dengue: Guidelines for Diagnosis, Treatment, Prevention and Control.* New Edition. Geneva:2009. [cited 2023 Feb 7]. Reference Source 23762963

[19] Lee VJ Lye DC Sun Y : Decision tree algorithm in deciding hospitalization for adult patients with dengue haemorrhagic fever in Singapore. *Trop. Med. Int. Health.* 2009 Sep;14(9):1154–1159. 10.1111/j.1365-3156.2009.02337.x 19624479

[20] Tamibmaniam J Hussin N Cheah WK : Proposal of a Clinical Decision Tree Algorithm Using Factors Associated with Severe Dengue Infection. *PLoS One.* 2016 Aug 1 [cited 2023 Feb 12];11(8):e0161696. 10.1371/journal.pone.0161696 27551776 PMC4994952

[21] Phakhounthong K Chaovalit P Jittamala P : Predicting the severity of dengue fever in children on admission based on clinical features and laboratory indicators: application of classification tree analysis. *BMC Pediatr.* 2018 Mar 13 [cited 2023 Feb 12];18(1):109. 10.1186/s12887-018-1078-y 29534694 PMC5850907

[22] Mahabala C Boloor A Upadhya S : Postural fall in Systolic Blood Pressure is an useful warning sign in Dengue Fever. *Dryad.* 10.5061/dryad.jwstqjqfc PMC10765096 38178940

