# Supplementary material for: Postural fall in systolic blood pressure is a useful warning sign in dengue fever
Source: F1000Res. 2023 Dec 18;12:816. Originally published 2023 Jul 11. [Version 2] doi: 10.12688/f1000research.132714.2 (PMC10765096; doi:10.12688/f1000research.132714.2)
Supplement: Peer review history

# Reviewer report: Reviewer response for version 2

**DOI:** 10.5256/f1000research.159870.r231032

**Published:** 2024-01-03

**Authors:** Soumya Pahari

**Affiliations:** 1 Nepalese Army Institute of Health Sciences (NAIHS), Sanobharyang, Nepal

I have no further comments to make.

Is the work clearly and accurately presented and does it cite the current literature?

Yes

If applicable, is the statistical analysis and its interpretation appropriate?

I cannot comment. A qualified statistician is required.

Are all the source data underlying the results available to ensure full reproducibility?

Yes

Is the study design appropriate and is the work technically sound?

Yes

Are the conclusions drawn adequately supported by the results?

Partly

Are sufficient details of methods and analysis provided to allow replication by others?

Partly

Reviewer Expertise:

Neurosurgery, Primary care medicine

I confirm that I have read this submission and believe that I have an appropriate level of expertise to confirm that it is of an acceptable scientific standard.

# Reviewer report: Reviewer response for version 2

**DOI:** 10.5256/f1000research.159870.r231031

**Published:** 2023-12-23

**Authors:** Rivaldo Steven Heriyanto

**Affiliations:** 1 Pelita Harapan University, Tangerang, Indonesia

The revision by the author(s) is sufficient, the article can proceed to the next step. Thank you for the opportunity.

Is the work clearly and accurately presented and does it cite the current literature?

Yes

If applicable, is the statistical analysis and its interpretation appropriate?

Partly

Are all the source data underlying the results available to ensure full reproducibility?

Yes

Is the study design appropriate and is the work technically sound?

Yes

Are the conclusions drawn adequately supported by the results?

Yes

Are sufficient details of methods and analysis provided to allow replication by others?

Yes

Reviewer Expertise:

Pediatric, Opthalmology, COVID-19, General

I confirm that I have read this submission and believe that I have an appropriate level of expertise to confirm that it is of an acceptable scientific standard.

# Reviewer report: Reviewer response for version 1

**DOI:** 10.5256/f1000research.145656.r220352

**Published:** 2023-11-14

**Authors:** Rivaldo Steven Heriyanto

**Affiliations:** 1 Pelita Harapan University, Tangerang, Indonesia

An overall well written article. However, a further grammar check is advised.

 The introduction is concise and explain the importance of analyzing the role of postural hypotension as dengue warning signs well, it is also a novel variable that should be researched further.

 The methods is also explained well, starting from type of studies, inclusion and exclusion criteria, sample size, to the explanation of the statistical analysis. The usage of CHAID method to make a decision tree model is an excellent decision that differentiate this study from other dengue warning sign research.

 The results answered the question of the research, starting from the adjusted odds ratio, the optimal cut-off of fall in SBP, up until the diagnostic accuracy of the decision tree. However, there is one comment, why do the author think that the result of the multiple logistic regression analysis resulted in only ascites/pleural effusion and fall in postural SBP that reached significant value? Is it possible that there is some biases that played some role?

 The discussion discusses the results well, explaining the importance of the results, as well as how this study compare to other. One comment is related to the result, the author should explain why the other warning signs doesn't have a significant value other than ascites/pleural effusion and fall in postural SBP

Is the work clearly and accurately presented and does it cite the current literature?

Yes

If applicable, is the statistical analysis and its interpretation appropriate?

Partly

Are all the source data underlying the results available to ensure full reproducibility?

Yes

Is the study design appropriate and is the work technically sound?

Yes

Are the conclusions drawn adequately supported by the results?

Yes

Are sufficient details of methods and analysis provided to allow replication by others?

Yes

Reviewer Expertise:

Pediatric, Opthalmology, COVID-19, General

I confirm that I have read this submission and believe that I have an appropriate level of expertise to confirm that it is of an acceptable scientific standard, however I have significant reservations, as outlined above.

# Response

**Published:** 2023-11-19

**Authors:** Archith Boloor

**Affiliations:** Medicine, Manipal Academy of Higher Education, Manipal, Karnataka, India

WHO has developed a set of clinical warning Symptoms for triaging patients to decide inpatient management. These 7 parameters have been studied in various studies to identify the relative importance of each one of them in predicting severe dengue. Tamibmaniam et al(1) have studied 657 patients with dengue of whom 59 had severe dengue. Even though many parameters were associated with severe dengue in simple logistic regression, adjusted odds ratios were significant for 3 parameters only after multiple logistic regression analysis (Fluid accumulation, vomiting and hypotension). Other parameters did not reach statistical significance in this multiple logistic regression. Our findings are also in line with these observations. It appears that some of the parameters seem to be having stronger independent association compared to other parameters. Our findings suggest that Postural fall in Systolic Blood Pressure and Pleural effusion/Ascites have much stronger independent associations with severe dengue, compared to other warning signs, resulting in relatively lower significance for other warning symptoms. Hence, these findings are unlikely to be due to bias. 

 1.         Tamibmaniam J, Hussin N, Cheah WK, Ng KS, Muninathan P. Proposal of a Clinical Decision Tree Algorithm Using Factors Associated with Severe Dengue Infection. PLoS One [Internet]. 2016 Aug 1 [cited 2023 Feb 12];11(8). Available from: https://pubmed.ncbi.nlm.nih.gov/27551776/ 

# Reviewer report: Reviewer response for version 1

**DOI:** 10.5256/f1000research.145656.r204024

**Published:** 2023-09-19

**Authors:** Soumya Pahari

**Affiliations:** 1 Nepalese Army Institute of Health Sciences (NAIHS), Sanobharyang, Nepal

First, I would like to congratulate the authors for their work in helping to improve and simplify the prediction of severe dengue.

1. Introduction/ Methods: Mention the working definition of “severe  dengue”.
2. Inclusion/ exclusion criteria: Please state and justify if patients with pre-existing postural hypotension were excluded from the study or not.
3. Sample size: Provide appropriate references for the expected prevalence of dengue being  15%. 
4. The presence of postural hypotension is assessed only at admission. Since the duration of fever at admission is approximately 4 days in the sample, the majority of them are likely to be at the febrile stage of illness and not entered the critical phase, where more frequent occurrences of plasma leaks and postural hypotension might have occurred. Was postural hypotension assessed for in the subsequent days of admission?

Is the work clearly and accurately presented and does it cite the current literature?

Yes

If applicable, is the statistical analysis and its interpretation appropriate?

I cannot comment. A qualified statistician is required.

Are all the source data underlying the results available to ensure full reproducibility?

Yes

Is the study design appropriate and is the work technically sound?

Yes

Are the conclusions drawn adequately supported by the results?

Partly

Are sufficient details of methods and analysis provided to allow replication by others?

Partly

Reviewer Expertise:

Neurosurgery, Primary care medicine

I confirm that I have read this submission and believe that I have an appropriate level of expertise to confirm that it is of an acceptable scientific standard, however I have significant reservations, as outlined above.

# Response

**Published:** 2023-09-28

**Authors:** Archith Boloor

**Affiliations:** Medicine, Manipal Academy of Higher Education, Manipal, Karnataka, India

1. WHO definition of severe dengue:

- Severe plasma leakage, leading to fluid accumulation with respiratory distress or shock
- Severe organ impairment (including cardiac, liver: ALT>1000 and CNS: altered consciousness)
- Severe bleeding.

2, Patients with pre-existing postural hypotension were excluded.

 3. In a previous study published by Alok kumar et al. 15.3% of hospitalised patients developed severe dengue (190/1234). Ref is given below:Alok Kumar, Shemica Mayers, Janelle Welch, Janine Taitt, Gemma Ann Benskin, Anders L. & Nielsen (2020) The spectrum of disease severity, the burden of hospitalizations and associated risk factors in confirmed dengue among persons of all ages: findings from a population based longitudinal study from Barbados, Infectious Diseases, 52:6, 396-404, DOI: 10.1080/23744235.2020.1749723

 4.Patients developing postural hypotension during the stay in the hospital were also included in the group with postural hypotension if they did not have clinical and laboratory features of severe dengue.
